# Cutaneous Metastasis of Bladder Cancer: A Systematic Review

**DOI:** 10.7759/cureus.74439

**Published:** 2024-11-25

**Authors:** Nagham Bazzi, Sadek Jaber, Wajih A Saad, Sally Al Hassan, Samer Dbouk, Ali Alhousseini

**Affiliations:** 1 Department of Medicine, Baabda Governmental Hospital, Beirut, LBN; 2 Department of Orthopedics, Lebanese University Faculty of Medicine, Beirut, LBN; 3 Department of Oncology, Lebanese University Faculty of Medicine, Beirut, LBN; 4 Department of Internal Medicine, American University of Beirut Medical Center, Beirut, LBN; 5 Department of General Surgery, Al Zahraa Hospital University Medical Center, Beirut, LBN; 6 Department of Obstetrics and Gynecology, Beaumont Hospital, Dearborn, USA

**Keywords:** bladder cancer, bladder metastasis, cutaneous metastasis, upper urinary tract cancer, uro oncology

## Abstract

Bladder cancer is one of the main causes of urogenital cancer (30-35% of the total urological cancers). Although metastases from urologic tumors are rare, it is associated with a high mortality rate. The location and pattern of metastasis are random and unpredictable. A literature search was done, and a total of 49 studies published from 1949 to 2021 were included. All published studies were case reports. A total number of 59 patients were included: 50 men and nine women. Multiple types of bladder cancer were described, 72.8% were urothelial/transitional cell carcinoma of different grades and stages (n=43). Other reported cancers were adenocarcinoma of the bladder (n=4), poorly differentiated carcinoma (n=5), bladder sarcoma (n=1), sarcomatoid carcinoma (n=1), renal cell carcinoma (n=1), myeloid sarcoma (n=1), and grade III papillary carcinoma (n=2). The most common site for cutaneous lesions was the abdomen, followed by the chest. Further randomized cohort studies assessing the sites and patterns of metastasis should be done to help physicians in their clinical work.

## Introduction and background

Metastasis is the most ominous and dreaded stage of cancer progression with a devastating prognosis and high mortality rate. It encompasses a spectrum of multistep biological processes leading to tumor transformation, a cornerstone in seed-soil implantation [1,2]. Fortunately, metastasis only occurs in a low percentage of cancers with the ability to overcome these inordinate biological boundaries. Cutaneous metastasis of bladder cancer is an uncommon phenomenon that induces a significant implication. The cancer can spread to distant sites through the bloodstream or lymphatics. Urogenital cancers have a vanishingly low propensity for cutaneous metastasis. Despite the dawning of new cancer treatment modalities in recent decades, a perversely counter-intuitive global figure of cutaneous metastasis originating from urological cancers has risen. However, the complex genomic landscape underlying tumor seeding is not fully elucidated yet [3].

This systematic review aims to provide a comprehensive overview of this rare manifestation. The review states various aspects of cutaneous metastasis of bladder cancer including incidence, clinical presentation, diagnostic methods, and treatment options. This systemic review will summarize the recently reported cases of urological cancer with skin metastasis, the proposed underlying pathophysiology, and the current knowledge of urogenetic metastasis.

Before conducting this systemic review, our hypothesis was that cutaneous metastasis of bladder cancer is uncommon and frequently denotes an advanced disease stage. We anticipated that the review would reveal a variety of clinical presentations, posing the diagnostic challenges and the underlying mechanisms that will give us an advantage in choosing the effective treatment for this rare manifestation.

## Review

Materials and methods

The systematic review was performed in accordance with the Preferred Reporting Items for Systematic Reviews and Meta-Analyses (PRISMA) guidelines [4]. A literature search in February 2022 was conducted and focused on cutaneous metastasis from bladder cancer. The databases were searched using the following query: "cutaneous metastasis from bladder cancer", "skin and bladder cancer", "skin metastasis and bladder cancer", and "transitional cell cancer and cutaneous metastases". Articles were evaluated by the authors and the most relevant articles were selected according to their titles and abstract. All articles published before the search date and pertaining to the topic were included. Systematic reviews, literature reviews, in vitro studies, articles not assessing the efficacy of xenografts, articles not written in English, articles conducted on animals, letters to the editor, and commentaries were excluded. For each included article, a reference list was extracted, and additional articles were identified and included.

Results

A total of 334 articles from 49 studies (with 59 patients) published between 1949 and 2021 were included in the study [5-56]. All included studies were case reports. Twelve studies were done in the United States, seven in Japan, five in India, four in Italy, and four in the United Kingdom. The rest of the included articles were published from several countries with a total of less than three articles from each country. Most of the studies included one patient except for seven studies that recruited two patients each [5-11]. A study published by Bischoff and Fishkin included four patients [12]. 

Patient Characteristics, History, and Laboratory Findings

Patient characteristics and past medical history: A total of 84.7% of the included patients were men (n=50) and 15.2% were women (n=9). Patients’ ages ranged between 33 and 88 years. Patients’ past medical history was not mentioned in almost half of the reports. Four patients had malignancies not related to the genito-urinary tract: superficial esophageal cancer, Hodgkin lymphoma, and two laryngeal cancers [13-16]. The most common urinary symptom was hematuria (47.4%, n=28) (Table 1).

**Table 1:** Summary of the Extracted Data From the Included Studies CR: clinical research; PMH: past medical history; UTI: urinary tract infection; NR: not recorded; HTN: hypertension; M: male; F: female; BPH: benign prostatic hyperplasia.

Study (year)	Country	Study design (level of evidence)	Total number of patients	Gender	Age (years)	PMH not related to urinary tract	PMH related to the urinary tract	Laboratory tests (abnormal)	Urinary tests	Positive immunohistochemical markers
Giunchi et al. (2017) [5]	Italy	CR	1	M	69	NR	Hematuria	NR	NR	GATA3, CK7, CK20, CK14
			1	M	71	NR	Hematuria	NR	NR	GATA3, CK7
Kumar et al. (2006) [6]	India	CR	1	M	55	NR	Hematuria	NR	NR	NR
			1	M	49	NR	NR	NR	NR	NR
Fujita et al.(1994) [7]	Japan	CR	1	M	77	NR	NR	NR	NR	NR
			1	M	72	NR	Pollakiuria, pain on urination	NR	NR	NR
Rebelakos et al. (1989) [8]	UK	CR	2	M	NR	NR	NR	NR	NR	NR
Bachrach and Dahlen (1973) [9]	USA	CR	1	M	77	NR	Dysuria	NR	NR	NR
			1	M	47	NR	NR	NR	NR	NR
Higgins and Hausfeld (1948) [10]	USA	CR	1	M	52	Normal	Pain on urination, intermittent frequency and nocturia, hematuria	NR	Heavy trace of albumin, RBC	NR
			1	M	51	Mastoidectomy, right inguinal hernia	Pain on urination, nocturia, hematuria, dysuria	NR	RBC, WBC	NR
McCrea (1949) [11]	USA	CR	1	M	71	NR	Painless hematuria	NR	NR	NR
			1	F	54	NR	Dysuria, hematuria	NR	NR	NR
Bischoff and Fishkin (1956) [12]	USA	CR	1	M	58	NR	Gross hematuria	NR	RBC, WBC	NR
			1	M	58	NR	Hematuria, frequency, burning dysuria	NR	RBC, WBC, albumin	NR
			1	M	55	TB	Hematuria, dysuria, retention	NR	RBC, albumin, WBC	NR
			1	M	76	NR	Hematuria, dysuria, urgency	NR	Albumin, RBC	NR
Yajima et al. (2021) [13]	Japan	CR	1	M	70	Superficial esophageal cancer	Gross hematuria	NR	Urine cytology: HGUC	CK7, CK20, and GATA 3
Blalock et al. (2011) [14]	USA	CR	1	M	80	Hodgkin lymphoma	NR	NR	NR	CK7, CD20, CK19, CD138
Wormer et al. (1956) [15]	USA	CR	1	M	88	Laryngeal carcinoma	Gross hematuria, nocturia, urgency	Normal	Albumin, RBC	NR
Cohen et al. (2017) [16]	USA	CR	1	M	75	Laryngeal cancer, depression, glaucoma, seizure, arthritis, thyroid disease, HTN, diabetes, GI hemorrhage	Gross hematuria with clots, frequency, nocturia, urgency, and dysuria	Hgb 5 g/dl	NR	Pan CK, CK7
Garg et al. (2018) [17]	India	CR	1	M	62	Decreased appetite	Oliguria	Hgb 7.2 g/dl, BUN 220 mg/dl, serum creatinine 6.5 mg/dl, K+ 6.5 meq/l, pH=7.2	NR	NR
Lees (2015) [18]	USA	CR	1	M	81	NR	NR	Hgb 10.3 g/dl, iron 3 umol/L, TSH 0.19 mU/L, creatinine 1.32 mg/dl	NR	CK5/6 and P63
Agarwal et al. (2014) [19]	India	CR	1	M	49	NR	Hematuria	Serum creatinine (2.2 mg/dl), BUN (23 mg/dl), K+(5.9 mg/dl), LDH (429 U/L)	RBC	CK7, CK20, keratin cocktail (AE1/A3), p63
Salemis et al. (2011) [20]	Greece	CR	1	M	68	NR	NR	Hgb (7.6 mg/dl), CRP (161 mg/dl), ALP (175 U/L), ESR (137 mm/h)	RBC	CEA, CA19-9
Gowardhan et al. (2004) [21]	UK	CR	1	M	51	NR	NR	NR	RBC	NR
Endo et al. (2021) [22]	Japan	CR	1	F	74	NR	NR	NR	Urine cytology: tumor cells	CK7+, CK20+
Schmiedecke et al. (2014) [23]	USA	CR	1	F	80	Ulcerative colitis, total colectomy, and ileostomy	Hematuria	Normal	Normal	CK7, CK20, p63, uroplakin III
Roos and Wigg (1993) [24]	Austria	CR	1	M	75	NR	Hematuria	Elevation ALP, LDH	Normal	NR
Stolla et al. (1994) [25]	France	CR	1	M	58	NR	Macroscopic hematuria	NR	NR	NR
Ghalleb et al. (2019) [26]	Tunisia	CR	1	M	61	NR	Hematuria	Hgb 10 g/dl	NR	CK7, P63
Miyamoto et al. (2000) [27]	Japan	CR	1	M	75	NR	NR	NR	NR	NR
Saito (1998) [28]	Japan	CR	1	M	79	NR	Asymptomatic hematuria	NR	NR	NR
Swick and Gordon (2010) [29]	USA	CR	1	M	69	NR	NR	NR	NR	Pan-keratin, CK7, CK20
Chin et al. (2011) [30]	Malaysia	CR	1	F	70	Diabetes, HTN, MDS	Frank hematuria	NR	NR	MPO
Hamer and Nourse (1953) [31]	USA	CR	1	M	71	BPH	Frequency, urgency, painful urination, hematuria	NR	NR	NR
Mathew et al. (2021) [32]	India	CR	1	M	76	Diabetes	Urinary symptoms without hematuria or dysuria	Normal	NR	Pan CK, CK7, CK20, and GATA 3
Hasan et al. (2020) [33]	USA	CR	1	F	71	NR	No	NR	NR	P63, P40, CK7, CK20, uroplakin
Kerkeni et al. (2017) [34]	Tunisia	CR	1	M	74	NR	NR	NR	NR	CK p63
Elumalai et al. (2020) [35]	India	CR	1	M	62	NR	NR	NR	NR	NR
Pomara et al. (2004) [36]	Italy	CR	1	M	76	NR	NR	NR	NR	NR
Manzelli et al. (2011) [37]	Italy	CR	1	M	68	NR	Gross hematuria	NR	NR	CK7, Cerb-B2
Zwenzner et al. (2006) [38]	Germany	CR	1	M	68	NR	NR	NR	NR	NR
Takematsu et al. (1984) [39]	Japan	CR	1	F	60	NR	NR	NR	NR	NR
Yoshida et al. (2012) [40]	Japan	CR	1	F	65	NR	NR	NR	NR	NR
Wyldes and Osborn (1988) [41]	UK	CR	1	M	43	NR	NR	NR	NR	NR
Yang et al. (2015) [42]	China	CR	1	M	53	NR	NR	NR	NR	CAM5.2, CDX2, CA199
Atmaca et al. (2007) [46]	Turkey	CR	1	M	66	NR	NR	NR	NR	LMW CK, HMFG-1
Akman et al. (2003) [47]	Turkey	CR	1	M	78	Normal	Hematuria	Inconclusive	NR	NR
Chang et al. (2013) [48]	China	CR	1	M	72	NR	Weak urine stream and dysuria	NR	NR	CK7, CK20
Chung et al. (2013) [49]	Korea	CR	1	M	60	NR	NR	Normal	NR	NR
Filho et al. (2016) [50]	Brazil	CR	1	M	63	Intermittent abdominal pain	UTI, hematuria	NR	NR	CK AE1/AE3, antivimentin
Somani et al. (2006) [52]	UK	CR	1	F	83	NR	Gross hematuria	NR	NR	CK7, CK20
Sgambato et al. (2009) [53]	Italy	CR	1	F	72	NR	NR	NR	NR	NR
Kumar et al. (2000) [54]	Iran	CR	1	M	58	NR	Hematuria and dysuria	NR	NR	NR
Feldmann et al. (2020) [55]	Austria	CR	1	M	78	NR	NR	NR	NR	CK7, CK20, and GATA 3
Salmanpoor et al. (2013) [56]	Iran	CR	1	M	33	NR	NR	NR	NR	CK (AE1/AE3), CK7, CK20, EMA, vimentin

Laboratory findings: Laboratory findings were not reported for most patients (77.9%, n=46). The most reported laboratory finding was anemia (8.4%, n=5). Elevated creatinine was reported in three patients [17-19]. The most common finding in urinalysis was the presence of RBCs seen in 16.9% of patients (n=10) [19-21]. Albumin was found on urinalysis in five patients and WBCs in four patients [10-12]. Malignant cells were found on urine cytology in two cases [13,22], and normal urinalysis was seen in two cases [23,24]. However, for the remaining 76.2% of patients, no urinalysis results were mentioned in the original papers (n=45). Additionally, the most common immunohistochemical marker was CK7 positive in 28.8% of patients (n=17) followed by CK20 in 20.3% of patients (n=12). Then, GATA3 and P63 were positive in five and six patients, respectively. The immunohistochemical studies were either not done or missing in 34 papers. 

*Characteristics of Skin Cancer* 

Types of urinary tract cancer, lymph node invasion, and non-cutaneous metastasis: Multiple types of bladder cancer were described in the different cases exposed in this paper. The vast majority, 72.8%, were urothelial/transitional cell carcinoma of different grades and stages (n=43). Other reported cancers were adenocarcinoma of the bladder (n=4), poorly differentiated carcinoma (n=5), bladder sarcoma (n=1), sarcomatoid carcinoma (n=1), renal cell carcinoma (n=1), myeloid sarcoma (n=1), and grade III papillary carcinoma (n=2). The type of bladder cancer was not reported in only one patient [25].

Interestingly, 12% of patients (n=7) had no lymph node involvement [16,18,21,26-28], whereas 45.7% of patients had an extension to lymph nodes (n=27) suggested mostly by enlarged lymph nodes on physical examination or on imaging, with the most involved nodes being inguinal and iliac. For the rest of the patients, data concerning the lymph nodes were not reported. Metastases to organs other than the skin were reported in the original articles in half of the patients (n=29), and only four patients had no metastasis to non-cutaneous organs [16,21,29,30]. The most common sites of metastasis were the lungs (n=11) followed by the liver (n=9), bone (n=5), and adrenals (n=5). Other mentioned sites of metastasis included the prostate (n=2), ureter (n=3), peritoneal carcinomatosis (n=3), the brain (n=2), the GI tract (n=4), and pericardium (n=2) (Table 2).

**Table 2:** Clinical Data Extracted From Individual Studies NR: not recorded; TCC: transitional cell carcinoma.

Study (year)	Total number of patients	Type of bladder cancer	Lymph node invasion	Non-cutaneous tumor metastasis	Skin lesion location	Cutaneous symptoms	Biopsy location	Biopsy finding	Date of skin manifestation occurrence	Non-cutaneous associated symptoms
Mathew et al (2021) [32]	1	Urothelial carcinoma	Enlarged lymph nodes along the bilateral iliac vessels	NR	The upper abdomen, back, and the scalp (above the right ear)	Firm, non-tender skin nodules	Abdominal wall nodule	Atypical pleomorphic cells with round nuclei, coarse chromatin, conspicuous nucleoli, and a moderate amount of cytoplasm with perineural invasion	At the time of diagnosis	Proptosis of the left eye
Yajima et al. (2021) [13]	1	Invasive high-grade urothelial carcinoma	Multiple lymph node metastases in the pelvis, enlarged para-aortic lymph nodes	Prostate	Left chest skin	Reddish mass on the skin	Cutaneous mass	Small nests of tumor cells in the dermis	2 months after detection of bladder cancer	NR
Endo et al. (2021) [22]	1	Renal pelvic urothelial carcinoma UC	Right neck, mediastinum, and para-aortic	NR	Neck and chest	Purpuritic, indurated plaque	NR	Nests of round, atypical cells with frequent tumor cell invasion into blood vessels and vascular destruction	1 month before detection of bladder cancer	Tracheal shifting and stenosis
Elumalai et al. (2020) [35]	1	Metastatic bladder carcinoma	NR	Osteolytic skeletal lesions, right 11th rib posteriorly	Anterior pelvic wall, walls of the scrotum, and base of the penis	Nodular thickening of cutaneous and subcutaneous tissue and ulcerations	Ulcerative lesion from the base of the penis	Nuclear atypia and lymphovascular emboli	8 years after diagnosis of primary urinary bladder carcinoma	NR
Feldmann et al. (2020) [55]	1	Transitional cell carcinoma of the bladder	The left inguinal region, single lymph node	NR	Pitting edema of the lower extremities with cobblestone appearance, including the genital area and the lower abdomen. On the border of the edema, there were erythematous patches mimicking erysipelas.	Erythematous patches mimicking erysipelas on the borders of the lower limb edema	Erythematous skin area	Atypical cells with pleomorphic and hyperchromatic nuclei within dermal lymphatic vessels	2 years after diagnosis	Pitting edema of lower extremities, including genital area and lower abdomen, with a cobblestone appearance
Hasan et al. (2020) [33]	1	Uroepithelial carcinoma of the bladder	Significant right inguinal, iliac, and retroperitoneal lymphadenopathy	Adrenal mass, left ureter	Suprapubic extended to the umbilicus, perianal, medial gluteal clefts, and anterior lower pelvis	Large erythematous indurated plaque, interval size soft-tissue nodules, and nodular skin thickening	Left abdomen	NR	At the time of diagnosis	Left lower extremity edema, suprapubic and external genital edema
Ghalleb et al. (2019) [26]	1	Urothelial carcinoma of the bladder and renal pelvis	No	Left ureter, lung	Left hypochondriac, cervical, and back regions	Cutaneous masses	Left hypochondriac lesion	NR	1 year after diagnosis of bladder carcinoma	NR
Garg et al. (2018) [17]	1	Adenocarcinoma of the bladder	NR	Multiple rounded cannon ball opacities in the lung	Neck, axilla, and scapula	Multiple rounded firm swellings with soft, cystic, non-tender erythematous swelling, with overlying superficial skin necrosis.	NR	NR	3 months before diagnosis	NR
Cohen et al. (2017) [16]	1	High-grade urothelial cell carcinoma with submucosal invasion	No	No	Right inguinal region and scrotum	Nodular, fibrotic, and hemorrhagic nodules	NR	Large anaplastic cells	9 months after diagnosis	Altered mental status
Kerkeni et al. (2017) [34]	1	Non-metastatic muscle-invasive transitional cell bladder carcinoma	2 lymph nodes	Liver mets and peritoneal carcinosis	NR	Multiple disseminated pigmented and flat skin lesions with subcutaneous nodules	Chest lesion	Poorly differentiated metastatic carcinoma	4 months after surgery	NR
Filho et al. (2016) [50]	1	High-grade urothelial carcinoma with invasion of the muscle	NR	Multiple peritoneal nodules of irregular outline and heterogeneous enhancement	Infra-umbilical, midline, and suprapubic region	Infra-umbilical scar, erythematous-purpuric plaque, infiltrated, fistula with urine drainage at midline, infra-umbilical region and 2 erythematous, wine-colored nodules at midline, suprapubic region	Midline scar nodule	Atypical epithelioid and pleomorphic cells with numerous mitoses and areas of necrosis	1 month after diagnosis	Abdominal distention and pain
Lees (2015) [18]	1	High-grade transitional cell carcinoma of the bladder	No	Osteoblastic metastases in the bony pelvis	Left anterior thigh	Maculo-nodular rash	Thigh lesions	Poorly differentiated non-small-cell carcinoma	8 years after diagnosis	NR
Giunchi et al. (2017) [5]	1	Urothelial carcinoma of the bladder	No	NR	Penis foreskin and glans	Condyloma acuminata, several brownish warty fragments on normal foreskin	Penis	Superficially spreading lesion composed of papillae with fibro-vascular cores. Neoplastic cells with wide cytoplasms, round nuclei, and prominent nucleoli. Squamous-like, nuclear atypias and micro-infiltrative areas	7 months after diagnosis	NR
	1	High-grade urothelial carcinoma of the bladder, prostatic urethra, and membranous urethra	Abdominal lymph nodes	Multiple repetitive hepatic lesion 11 years after diagnosis	Penis glans	Reddish skin lesion	Penis	High-grade papillary urothelial carcinoma with infiltration of corpus spongiosum, large pale cells arranged in small nests above the basal layer with cleft-like separation between neoplastic cells and normal epidermal keratinocytes in the glans	10 years after diagnosis	NR
Yang et al. (2015) [42]	1	Urachal adenocarcinoma of the bladder with enteric-type differentiation, mucinous adenocarcinoma, and ring cell carcinoma	NR	NR	Penis and scrotum	2 cm diameter mass, 10 cm erysipeloid-like mass	Penis	Original and metastatic urachal tumor, well-differentiated mucinous adenocarcinoma, signet ring cells	2 months after diagnosis	NR
Schmiedecke et al. (2014) [23]	1	Transitional cell carcinoma of the bladder	Inguinal node	NR	Adjacent to RLQ ileostomy site	Nodular-appearing erythematous plaque	Ileostomy site	Cohesive nests of malignant cells in dermoplastic stroma	10 years after diagnosis	NR
Agarwal et al. (2014) [19]	1	Transitional cell carcinoma of the bladder	Peritoneal nodules, posterior abdominal wall, gluteal region, bilateral axillary, pelvic, and inguinal	Large liver mass, multiple right adrenal masses	Flank mass	Mass	Flank mass	Undifferentiated carcinoma with clear cell features	At the time of diagnosis	Fatigue, chronic renal insufficiency, balanitis
Salmanpoor et al. (2013) [56]	1	Urothelial carcinoma	NR	NR	Back and posterior neck	Extensive rapidly growing, indurated, firm violaceous erythematous tumor with ill-defined borders and woody induration	Back skin	Oval- to spindle-shaped nuclei, conspicuous nucleoli, ill-defined cell borders with mitotic figures	At the time of diagnosis	Free fluid in the peritoneal cavity, thickening of the wall of the fundus and body of the stomach
Chang et al. (2013) [48]	1	High-grade urothelial carcinoma	Para-aortic lymph node enlargement	Pulmonary mass and peribronchial lesions	Left flank area and suprapubic area, then on suprapubic area, chest, and abdomen	Palm-sized erythematous plaques, extensive painful erythematous plaques with an erysipelas-like appearance	Left chest and suprapubic area	Tumor nests in lymphatics with outgrowth into the surrounding dermis, with pleomorphic and hyperchromatic nuclei	1 month after diagnosis	NR
Chung et al. (2013) [49]	1	Urothelial carcinoma	Bilateral inguinal lymph nodes, both iliac chain lymph nodes	Lungs, brain	Abdominal skin	Multiple erythematic nodular lesions	Abdominal skin	Metastatic urothelial carcinoma	8 years after diagnosis	Severe dyspnea, dizziness
Yoshida et al. (2012) [40]	1	High-grade urothelial carcinoma with glandular differentiation	Pelvic lymph nodes	Ureter	Around the soma	Painless skin erosion	Skin lesion	Malignant cells suspected of being urothelial carcinoma	4 years and a half after diagnosis	NR
Manzelli et al. (2011) [37]	1	Sarcomatoid carcinoma invading bladder musculature	Loco-regional node enlargement	Lungs	Right chest wall	Hypodermic nodular lesion	Right chest wall	Epithelial-type cells with nuclear atypias, poorly differentiated neoplastic infiltration with morphologic aspects of urothelial tissue	At the time of diagnosis	NR
Blalock et al. (2011) [14]	1	Minimally invasive TCC	Lymphovascular invasion, inguinal and femoral lymphadenopathy	NR	Left lower abdomen	Firm pink plaque with "frog spawn", with small peripheral papules extending from the edge of the larger plaque	Abdomen	Full-thickness malignant epithelial neoplasm with nests of tumor cells involving the dermis and invading the epidermis	1 year and a half after diagnosis	NR
Salemis et al. (2011) [20]	1	High-grade TCC	NR	NR	Left lower abdomen	Hard non-tender palpable nodule	Abdomen	Extensive infiltration from high-grade transitional cell bladder carcinoma	1 year and 2 months after diagnosis	NR
Swick et al. (2010) [29]	1	Superficially invasive TCC	Periaortic and pelvic lymphadenopathy	No	Mid-upper back, left mid-back, lower thoracic spine	The asymptomatic lesion which began as a small flesh-colored nodule became violaceous	Larger nodule	Proliferation of atypical cells infiltrating in nests and cords, collagen bundles through the mid and deep-reticular dermis, no involvement of overlying epidermis, individual cells with abundant cytoplasm and hyperchromatic atypical nuclei with frequent mitoses	3 months after diagnosis	Acute-onset diplopia, persistent headaches
Sgambato et al. (2009) [53]	1	Bladder adenocarcinoma	NR	NR	Scalp and back	Red firm nodular lesions	Scalp	Epidermotropic tumor cells appear singly and in a cluster as polygonal, large, refractile structures embedded in the epidermis, at the dermal level, tumor nests composed of large, mildly refractile, polymorphic cells arranged in a "palisading" array around a luminal area filled with hyporefractile material.	7 years after diagnosis	NR
Atmaca et al. (2007) [46]	1	Superficial TCC	NR	Skeletal	Trunk and upper extremities	Multiple subcutaneous nodules	Nodules	TCC metastasis	3 months after diagnosis	NR
Zwenzner et al. (2006) [38]	1	TCC	Pelvic lymphadenopathy	NR	Neck	Fast-growing poorly demarcated erythematous induration	Neck	Multiple nests of urothelial cells, with cytologic atypia	3 years after diagnosis	NR
Kumar et al. (2006) [6]	1	Muscle invasive grade II TCC	Pelvic lymphadenopathy	NR	Right arm	Nodular swelling, slightly reddish, smooth-surfaced nodule with central crusting	Arm	Metastatic carcinoma from the bladder	After many cycles of chemotherapy	NR
	1	Renal cell carcinoma	NR	NR	Left postauricular area	Swelling and purple, smooth-surfaced non-tender nodule	Postauricular area	Metastatic carcinoma from kidney	4 months before diagnosis	Abdominal pain
Pomara et al. (2004) [36]	1	TCC	Enlarged pelvic lymph nodes	Upper right pulmonary lobe	Penis glans	Ulcerative swelling, fungating lesion with seropurulent discharge, partially ulcerated, erythematous, and slightly itchy	Glans	Urothelial carcinoma	8 years after diagnosis	NR
Gowardhan et al. (2004) [21]	1	Extensive muscle-invasive bladder TCC	No	No	Left iliac fossa	Skin nodule	Nodule	Extensive infiltration of the dermis and subcutis by high-grade urothelial carcinoma	3 months after diagnosis	NR
Akman et al. (2003) [47]	1	Poorly differentiated carcinoma	NR	Liver	Trunk	Multiple, rubbery subcutaneous nodules	Trunk	Metastasis of TCC	3 months after diagnosis	NR
Somani et al. (2006) [52]	1	Poorly differentiated, muscle-invasive TCC with carcinoma in situ	NR	NR	Left side of neck and chest	Herpetiform-like, multiple reddish nodules	Lesions	Metastatic carcinoma similar to TCC of the bladder	6 weeks after diagnosis	NR
Miyamoto et al. (2000) [27]	1	Poorly differentiated grade III TCC	No	NR	Penis	Pseudophimosis, a reddish oval tumor on corona glandis, well-demarcated erythema with slight erosion on preputium and glans	Penis	Nest of atypical malignant cells on the surface	3 years after diagnosis	NR
Kumar et al. (2000) [54]	1	Grade IV TCC	Enlarged para-aortic lymph nodes	NR	Right lumbar paraspinal region	Subcutaneous mass, painful, soft, and nonmobile	Paraspinal	Isolated and clusters of malignant cells, different sizes and shapes, high nuclear/cytoplasmic ratios, hyperchromatic nuclei with prominent nucleoli and abundant, deep, basophilic cytoplasm, plus frequent multinucleated tumor giant cells. Clusters of different sizes and shapes, irregular and papillary in configuration	6 months after diagnosis	NR
Saito (1998) [28]	1	Grade II TCC	No	NR	Scrotum	Solitary subcutaneous induration, firm, moderately tender	Left testis	TCC metastasis	1 year and a half after diagnosis	NR
Stolla et al. (1994) [25]	1	NR	4th and 7th nodes	Liver	Celioscopy puncture scars	NR	Scar	Permeation nodules of a poorly differentiated carcinoma	9 months after diagnosis	NR
Roos and Wigg (1993) [24]	1	Grade II TCC	Left supraclavicular lymphadenopathy	NR	Left shoulder	Diffuse nodular, violaceous tumor with well-demarcated borders, with C6 neuralgia	Left shoulder	Metastasis of TCC	1 year after diagnosis	Anorexia, weight loss
Fujita et al.(1994) [7]	1	TCC	Cervical and para-aortic lymphadenopathy	NR	Thighs and lower abdomen	Erysipeloid skin rash	Thigh and cheek	Metastatic TCC	3 months after diagnosis	NR
	1	Grade III TCC	Para-aortic lymph nodes enlargement, Virchow nodes, retroperitoneal parapancreatic, mediastinal	Pleura of lungs, liver, bones, GI tract	Thighs and lower abdomen	Erythematous skin eruptions	Thigh	Metastatic TCC	2 months after diagnosis	NR
Rebelakos et al. (1989) [8]	2	TCC	NR	NR	Left mid sternum	Painful nodule	Sternum	TCC metastasis	2 months after diagnosis	NR
Wyldes and Osborn (1988) [41]	1	Solid, poorly differentiated TCC	NR	NR	Right forearm	Painful swelling, firm, moderately tender, not fluctuant, with raised well-demarcated edge, surrounding skin indurated with marked erythema	Forearm	Small group of malignant cells having the appearance of original bladder tumor	3 months after diagnosis	NR
Takematsu et al. (1984) [39]	1	Adenocarcinoma of the bladder	NR	Lower GI, pelvic wall, liver and spleen	Right inguinal region to the middle right thigh	Papillomatous nodule, with cauliflower-like proliferation with a large quantity of foul-smelling exudate	Nodule	Well-differentiated adenocarcinoma	At the time of diagnosis	NR
Bachrach and Dahlen (1973) [9]	1	Poorly differentiated TCC	NR	Brain, penis	Neck and abdominal wall	Ulcerated lesions	Subcutaneous skin nodule	Anaplastic poorly differentiated metastatic carcinoma with areas of necrosis	At the time of diagnosis	Personality change, priapism, weight loss, left hemiparesis
	1	Bladder carcinoma	NR	Scalp, lungs, and retina	The right shaft of the penis	Non-tender area of induration	Penis	Fibro-vascular tissue infiltrated by nests of malignant tumor cells consistent with metastatic carcinoma	2 months after diagnosis	NR
Higgins and Hausfeld (1948) [10]	1	Highly undifferentiated infiltrating carcinoma	Virchow nodes	NR	End of cystectomy incision, right biceps, left supraclavicular space, under right rib margin	Small lump, firm subcutaneous nodule, freely movable and slightly tender	NR	NR	2 years after diagnosis	NR
	1	TCC	Cervical lymphadenopathy	Liver, lungs, posterior peritoneum	Above left iliac crest, left posterior cervical region, abdomen, lower thorax. Left upper extremity	Pitting edema, hard superficial, adherent nodule	NR	Smooth surface, grayish-white color	At the time of diagnosis	Right groin pain, nausea, vomiting
Bischoff and Fishkin (1956) [12]	1	TCC	Iliac lymphadenopathy	Pericardium	Head, neck, arms, and chest	Subcutaneous nodules, hard, painless, movable with orange peel surface	Right chest wall	Infiltrating nests and cords of transitional epithelial cells (metastatic TCC)	1 year after diagnosis	Dyspnea, ankle edema
	1	Grade III TCC with adenocarcinoma features		Bone, liver, adrenals, small bowel, mesentery, lungs	Left deltoid and posterior axillary line over 7th rib	Elevated, dusky red, firm, tender, fixed nodule in subcutaneous tissue	Nodules	Loose cords of polyhedral tumor cells interspersed in a delicate fibrous stroma, containing many inflammatory cells, with finely granular cytoplasm	3 years after diagnosis	Weight loss
	1	TCC	NR	Pericardium, adrenals, pancreas, cerebellar hemisphere	Left arm, cystotomy scar, right axilla, left axilla, chest, abdomen, shoulders, thighs	Small lump, many nodules	Nodules	Undifferentiated carcinoma with clear cell features	At the time of diagnosis	Abdominal, left flank pain, pitting edema
	1	TCC	NR	NR	Mid-epigastrium	Ulcerative lesion	Lesion	Neoplastic involvement of all layers except the epidermis, cells polyhedral shape, containing vesicular nuclear patterns with prominent nucleoli, fibrovascular cores containing polymorphonuclear leukocytes. (metastatic TCC)	A few months after diagnosis	Weight loss, hypertension, left cornea opacification, left indirect inguinal hernia, bilateral spermatoceles, BPH
Wormer et al. (1956) [15]	1	Undifferentiated carcinoma	NR	NR	Left cheek, left periorbital tissues	Small red spot enlarging progressively and painlessly	Cheek	Undifferentiated carcinoma	1 year before diagnosis	NR
Hamer and Nourse (1951) [31]	1	Grade III undifferentiated infiltrating carcinoma	NR	Prostate, seminal vesicles	Lateral to ureterostomy incision	Subcutaneous nodules	Nodule	Metastatic carcinoma	1 month after diagnosis	Weight loss
McCrea (1949) [11]	1	Grade III papillary carcinoma	NR	Lung	Back and shoulders	Several painful nodules	Nodule	Cores and islands of relatively anaplastic cells embedded in hyalinized stroma, pseudo-acinar formation (urinary bladder primary carcinoma)	At the time of diagnosis	NR
	1	Grade III papillary carcinoma	Inguinal lymph nodes	Right ileum, lung, adrenals	Abdomen	Nodule	Abdomen	Streaks of atypical epithelial cells with large nuclei and vacuolated cytoplasm, very numerous mitotic figures (metastatic carcinoma)	At the time of diagnosis	NR
Chin et al. (2011) [30]	1	Myeloid sarcoma	Pelvic and inguinal lymphadenopathy	No	Suprapubic region	Hyperemic skin	Suprapubic region	Myeloid sarcoma	At the time of diagnosis	Abdominal pain

Presentation of cutaneous lesions: The most common site for cutaneous lesions was the abdomen (n=15) followed by the chest (n=12). Cutaneous metastasis on the upper extremities including the shoulders and axillae was present in 18.6% of patients (n=11) and on the back was found in 18.6% of patients (n=11). Neck cutaneous metastasis and inguinal region (including penis and scrotum) were present in 10 patients each. Less common sites of cutaneous spread were the pelvis (n=9), thigh (n=6), and head (scalp or face) (n=4). The site of cutaneous metastasis was not mentioned in only one report. It is noteworthy that lesions arose in areas of previous procedures in many reports: adjacent to ileostomy site [22], celioscopy puncture scars [24], cystectomy incision [9], cystotomy scar [11], lateral to ureterostomy scar [30]. The skin findings in the different reported cases were heterogeneous, but the most recurrent finding in most of the described cases was a nodular component. In some reports, the nodule was painful [7,10]; but in most of them, the nodule was painless [11,19,31]. In many cases, it was accompanied by another finding [32,33]. Moreover, in some cases, the cutaneous metastasis appeared simply as an erythematous flat lesion with no additional characteristics [4,6,14]. Ulceration was seen in some cases [8,34,35].

Cutaneous biopsy: In addition to the above-mentioned macroscopic descriptions, biopsies were performed and showed a wide variety of findings. As expected, nuclear and cellular atypias [36,37], as well as abundant mitoses, were frequently described [5-10]. The urothelial cells were well differentiated in some reports [38] and undifferentiated in others [8-14]. The different layers of the skin, epidermis, dermis, and subcutaneous tissue, were also invaded to variable degrees; in fact, the epidermis was spared in some cases [20,28] and invaded in others [13].

Date of skin manifestations: Out of the 59 patients with bladder cancer, 72.8% had the skin manifestation occurring after the bladder cancer diagnosis (n=43) by months or even years (up to 10 years in two case reports). Interestingly, in 6.7% of the patients (n=4), the skin lesion appeared before the bladder cancer diagnosis, and in the remaining 24.4%, the cutaneous manifestation was identified at the same time as the bladder cancer diagnosis (n=12). The earliest skin lesion identification was made one year before the bladder malignancy diagnosis [14]. Among the non-cutaneous associated symptoms, some of the most mentioned complaints were edema of the lower extremities and genital area (n=4), abdominal distention or pain (n=4), and weight loss (n=5). 

*Treatment and Prognosis* 

Treatment of cutaneous lesions: In some cases, the same treatment strategy used to treat urinary tract cancer was aimed at improving skin manifestations too. In other cases, different treatment modalities were added to address the skin lesions. In about 38% of the cases (n=23), the original article did not mention the treatment strategy used for the cutaneous lesions. In another 10% (n=6), no treatment was used for skin manifestations and it was mentioned that in one of these cases, the treatment modality was proposed to the patient but declined. Chemotherapy alone was used in the treatment of skin lesions in 16.9% of the cases (n=10), with some of the mentioned regimens being pembrolizumab (n=2), gemcitabine with cisplatin (n=1), gemcitabine with carboplatin (n=2), vinblastine (n=1), bleomycin ointment (n=1), and methotrexate (n=1). About 15% of cases (n=9) had treatment with radiotherapy with a special mention to Roentgen therapy in one report. Surgery for the organ with affected skin occurred in 8% of the cases (n=5) with resection of the glands in one case and resection of the penis, scrotum, and groin skin in another. A combination of chemotherapy and radiotherapy was used in two cases, resection and chemotherapy together were used in one case, and a combination of radiation and resection was also used in only one case. In addition, one article mentioned using electrotherapy and one article only went for supportive treatment of the skin (Table 3).

**Table 3:** Treatment and Prognosis Data From Individual Studies NR: not recorded; TCC: transitional cell carcinoma; TURBT: transurethral resection of bladder tumor.

Study (year)	Total number of patients	Type of bladder cancer	Treatment of cutaneous lesion	Treatment of bladder cancer	
Mathew et al. (2021) [32]	1	Urothelial carcinoma	NR	Palliative chemotherapy (cisplatin-based)	
Yajima et al. (2021) [13]	1	Invasive high-grade urothelial carcinoma	Chemotherapy (pembrolizumab)	Transurethral resection of tumor and adjuvant chemotherapy (cisplatin and gemcitabine)	
Endo et al. (2021) [22]	1	Renal pelvic urothelial carcinoma UC	NR	NR	
Elumalai et al. (2020) [35]	1	Metastatic bladder carcinoma	NR	Transurethral resection of the tumor, radiotherapy of the pelvis, and chemotherapy	
Feldmann et al. (2020) [55]	1	Transitional cell carcinoma of the bladder	Pembrolizumab	Transurethral resection of tumor, chemotherapy (gemcitabine and cisplatin)	
Hasan et al. (2020) [33]	1	Uroepithelial carcinoma of the bladder	NR	TURBT with palliative chemotherapy (cisplatin and gemcitabine)	
Ghalleb et al. (2019) [26]	1	Urothelial carcinoma of the bladder and renal pelvis	NR	TURBT with palliative chemotherapy (cisplatin and gemcitabine)	
Garg et al. (2018) [17]	1	Adenocarcinoma of bladder	None	Bilateral percutaneous nephrostomy tubes and hemodialysis	
Cohen et al. (2017) [16]	1	High-grade urothelial cell carcinoma with submucosal invasion	None	Multiple TURBTs and BCG immunotherapy	
Kerkeni et al. (2017) [34]	1	Non-metastatic muscle-invasive transitional cell bladder carcinoma	NR	Cysto-prostatectomy with chemotherapy (cisplatin-based adjuvant)	
Filho et al. (2016) [50]	1	High-grade urothelial carcinoma with invasion of the muscle	NR	TURBT with palliative chemotherapy (cisplatin and gemcitabine)	
Lees (2015) [18]	1	High-grade transitional cell carcinoma of the bladder	Palliative radiation of left thigh lesions	Cystectomy and ileostomy	
Giunchi et al. (2017) [5]	1	Urothelial carcinoma of the bladder	NR	NR	
	1	High-grade urothelial carcinoma of the bladder, prostatic urethra, and membranous urethra	Complete resection of glans	Radical cystectomy	
Yang et al. (2015) [42]	1	Urachal adenocarcinoma of the bladder with enteric-type differentiation, mucinous adenocarcinoma, and ring cell carcinoma	Total resection of penis, scrotum, and groin skin	Partial cystectomy	
Schmiedecke et al. (2014) [23]	1	Transitional cell carcinoma of the bladder	Declined	NR	
Agarwal et al. (2014) [19]	1	Transitional cell carcinoma of the bladder	NR	TURBT, chemotherapy (gemcitabine, cisplatin, carboplatin)	
Salmanpoor et al. (2013) [56]	1	Urothelial carcinoma	NR	NR	
Chang et al. (2013) [48]	1	High-grade urothelial carcinoma	Chemotherapy	Chemotherapy	
Chung et al. (2013) [49]	1	Urothelial carcinoma	NR	Cystectomy with ileal conduit, chemotherapy (gemcitabine and cisplatin)	
Yoshida et al. (2012) [40]	1	High-grade urothelial carcinoma with glandular differentiation	Chemotherapy (gemcitabine and cisplatin)	Cystectomy	
Manzelli et al. (2011) [37]	1	Sarcomatoid carcinoma invading bladder musculature	Surgical resection	TURBT, chemotherapy (gemcitabine, cisplatin, carboplatin, and paclitaxel)	
Blalock et al. (2011) [14]	1	Minimally invasive TCC	Chemotherapy (carboplatin and gemcitabine)	TURBT, BCG treatment	
Salemis et al. (2011) [20]	1	High-grade TCC	Supportive treatment	Radical cystectomy and bilateral ureterostomy	
Swick et al. (2009) [22]	1	Superficially invasive TCC	NR	TURBT with BCG	
Sgambato et al. (2009) [53]	1	Bladder adenocarcinoma	NR	Surgery and local chemotherapy	
Atmaca et al. (2007) [46]	1	Superficial TCC	Chemotherapy	TRUBT and intravesical mitomycin	
Zwenzner et al. (2006) [38]	1	TCC	None	Cystoprostatovesiculectomy and chemotherapy (gemcitabine, cisplatin, paclitaxel, and carboplatin)	
Kumar et al. (2006) [54]	1	Muscle-invasive grade II TCC	None	Chemotherapy (gemcitabine and cisplatin)	
	1	Renal cell carcinoma	Chemotherapy (vinblastine)	Chemotherapy (vinblastine)	
Pomara et al. (2004) [36]	1	TCC	Combination chemotherapy (gemcitabine) and radiotherapy	Nephro-ureterectomy	
Gowardhan et al. (2004) [21]	1	Extensive muscle-invasive bladder TCC	Oral methotrexate	TURBT, radical cystoprostatourethrectomy, and radiotherapy	
Akman et al. (2002) [47]	1	Poorly differentiated carcinoma	Chemotherapy (gemcitabine and carboplatin)	TURBT, chemotherapy (gemcitabine and carboplatin)	
Somani et al. (2006) [52]	1	Poorly differentiated muscle-invasive TCC with carcinoma in situ	Radiotherapy	TURBT, palliative radiotherapy	
Miyamoto et al. (2000) [27]	1	Poorly differentiated grade III TCC	5-fluorouracil and corticosteroid ointment then resection	Chemotherapy (cisplatin and doxorubicin)	
Kumar et al. (2000) [6]	1	Grade IV TCC	None	Radiotherapy and chemotherapy	
Saito (1998) [28]	1	Grade II TCC	Resection, chemotherapy (MVAC)	TURBT, bladder infiltration with BCG	
Stolla et al. (1994) [25]	1	NR	Electrotherapy	Endourethral resection, chemotherapy (MVAC), radiochemotherapy, with 5-fluorouracil and cis-platinum	
Roos et al. (1993) [24]	1	Grade II TCC	Palliative chemotherapy, radiotherapy	Radiotherapy	
Fujita et al.(1994) [7]	1	TCC	Radiotherapy	TURBT, tegafur	
	1	Grade III TCC	Ointment (bleomycin)	TURBT, radical cystectomy, nephro-urectomy, chemotherapy (cis-platinum, doxorubicin, cyclophosphamide), radiotherapy	
Rebelakos et al. (1989) [8]	2	TCC	Radiotherapy with excision (n=1)	NR	
			Radiotherapy without excision	NR	
Wyldes and Osborn (1988) [41]	1	Solid, poorly differentiated TCC	Radiotherapy	Pelvic radiotherapy	
Takematsu et al. (1984) [39]	1	Adenocarcinoma of bladder	NR	Chemotherapy (doxorubicin and fluorouracil)	
Bachrach and Dahlen (1973) [9]	1	Poorly differentiated TCC	NR	NR	
	1	Bladder carcinoma	Radiotherapy	Ileal conduit, radiotherapy	
Higgins and Hausfeld (1950) [10]	1	Highly undifferentiated infiltrating carcinoma	Radiotherapy	Bilateral ureterosigmoidostomy, cystectomy	
	1	TCC	NR	TURBT, radiotherapy	
Bischoff and Fishkin [12]	1	TCC	NR	NR	
	1	Grade III TCC with adenocarcinoma features	NR	NR	
	1	TCC	Roentgen therapy	Transvesical fulguration	
	1	TCC	NR	Segmental resection	
Wormer et al. (1956) [15]	1	Undifferentiated carcinoma	Radiotherapy	TURBT, radiotherapy	
Hamer and Nourse (1953) [31]	1	Grade III undifferentiated infiltrating carcinoma	NR	Cystectomy	
McCrea (1949) [11]	1	Grade III papillary carcinoma	NR	Radiotherapy, fulguration	
	1	Grade III papillary carcinoma	NR	NR	
Chin et al. (2011) [30]	1	Myeloid sarcoma	NR	TURBT, palliative chemotherapy (Ara C and Myclotarg)	

Treatment of bladder cancer: As for the treatment of primary urinary tract cancer, it was not mentioned for 16.9% of the cases (n=10). The most common strategy was a combination of surgery and chemotherapy in 23% of the cases (n=14) with cisplatin and gemcitabine being frequently used. Surgery alone was used in 18% of the cases (n=11), chemotherapy alone was used in 10% of the cases (n=6), and radiotherapy alone was used in 3% of the cases (n=2). Note that cystectomy was frequently performed (n=7) and other surgical approaches included transvesical fulguration, nephroureterectomy, segmental resection, and bilateral percutaneous nephrostomy tube placement with subsequent hemodialysis. As for chemotherapy modalities, these included cisplatin, cisplatin+gemcitabine, cisplatin+doxorubicin, vinblastine, and doxorubicin+fluorouracil. 

In addition, a combination of radiotherapy and surgery was seen in 10% of the cases, whereas a combination of radiotherapy, surgery, and chemotherapy was seen in 5% of the cases (n=3). Radiotherapy and chemotherapy together were used in only one case. Finally, it is worth mentioning that surgery with BCG injection was the treatment modality used in 8% of the cases (n=5). 

Prognosis: Death was the most reported outcome, seen in 33 patients, which is 55.9% of the cases. The date of death was reported heterogeneously in articles with some announcing the date of death from the biopsy date, while others from the cutaneous manifestation date and so on. However, most of the 33 patients survived only for a few months. No information about prognosis was found in 23% of cases (n=14). For the rest of the patients, remarkably, a good prognosis was mentioned in one case [14], and disease-free progression was seen at three months in one case [39], at four months in another [40], at seven months [41], two years [4], 15 months [27], four years [26], and 23 years [20].

Discussion

Skin cancer is the sixth most prevalent cancer in women and the fifth most common cancer in men [42]. The most common primary tumors of skin metastasis are breast cancer (69%) and lung carcinoma (24%), in women and men, respectively [43]. Colorectal carcinoma is the second most common primary malignancy sending skin metastasis in men and women [43]. Metastases from urologic tumors are rare, occurring in around 1% of individuals with advanced illness [41-43]. Lymph nodes, bones, and lungs are the most common organs with urologic metastasis [44,45]. The first cutaneous tumor originating from transitional cell carcinoma of the bladder was reported in 1909 [16].

There are four mechanisms by which visceral tumors can metastasize to the skin: direct invasion, iatrogenic, lymphatics, or hematologic [45-47]. Cutaneous metastasis from urologic conditions may clinically look similar to other prevalent dermatologic conditions that affect people with advanced malignancies. Thus, a skin biopsy and an index of suspicion are needed for a conclusive diagnosis [43].

Punch biopsy from the cutaneous lesions showed an increased mitotic activity with malignant cells in the dermis rather than the epidermis [48-51]. These lesions appear as rapidly growing solitary or multiple flesh-colored lesions, with a necrotic or ulcerated aspect and with erythematous surrounding skin [28,50,52,53]. These findings were similar to the reported data in our text. Primary tumors of the metastatic lesion can be identified histologically. Renal cell carcinoma appears as a pulsatile solitary red nodule [43].

The differential diagnosis is wide, especially for patients taking systemic treatment for bladder cancer. Therefore, the lesions might appear as a possible cutaneous drug reaction or an infected lesion from an opportunistic infection. The differential diagnosis might also include hemangiomas, keratoacanthomas, cysts, pyogenic granuloma, dermatofibroma, pyoderma, and morphea-like lesions herpes zoster and erysipelas [46-48,52,54,55-57]. 

## Conclusions

The most common metastasis of urogenital cancer occurs in the liver, lungs, and bones. Among the various urogenital cancers, bladder cancer is the most common and is the fourth most common malignancy in males and the tenth in females. Cutaneous metastasis of bladder cancer is extremely rare with an incidence of less than 1%. The disease occurs via direct tumor invasion, hematogenous lymphatic spread, or direct seeding due to an iatrogenic implantation.

This paper provides a deeper understanding of the characteristics of bladder cancer spread and would help physicians in their diagnosis and management. Further randomized cohort study assessing the spread of malignancy is now warranted.
